# Grandparenting Styles in the U.S.: Patterns of Intergenerational Support and Their Predictors

**DOI:** 10.1093/geroni/igaf122.1894

**Published:** 2025-12-31

**Authors:** Hyeonji Cho, Catherine Garcia

**Affiliations:** Syracuse University, Syracuse, New York, United States; Syracuse University, Syracuse, New York, United States

## Abstract

Diversity in aging and recent demographic changes could contribute to heterogeneous grandparenting patterns. We classified grandparenting styles in the U.S. based on intergenerational support dynamics and examined predictors of grandparenting style membership. Using data from the Health and Retirement Study (2012–2018), we analyzed 12,435 households where the respondent or their spouse had at least one child and grandchild. Latent class analysis was conducted on five intergenerational support indicators: providing financial support, providing grandchild care, living with children, receiving financial support, receiving ADL/IADL assistance. A three-class model provided the best fit. “Active Providers (28%)” provided both financial and childcare support while receiving minimal assistance from their children. “Financially Supportive but Detached (55%)” grandparents primarily offered financial support but had lower overall engagement. “Co-residing Recipients (17%)” were grandparents who live with their children and were more likely to receive both financial and instrumental support. Multinomial logistic regression examined predictors of grandparenting style membership, with “Financially Supportive but Detached” as a reference group. Higher household income (RRR=1.09, p<.001) and better health (RRR=1.17, p<.001) were associated with a greater likelihood of being in the “Active Providers” group, while lower household income (RRR=0.94, p<.001) and poorer health (RRR=0.55, p<.001) were associated with membership in the “Co-residing Recipients” group. Additionally, households with non-White respondents were more likely to be classified as “Active Providers” and “Co-residing Recipients” compared to White grandparents. Findings illustrate how financial resources, health, and race shape grandparenting roles. Recognizing these patterns can help inform policies and programs for aging families and multigenerational households.

